# Elevated meteorin-like protein from high-intensity interval training improves heart function via AMPK/HDAC4 pathway

**DOI:** 10.1016/j.gendis.2023.101100

**Published:** 2023-09-14

**Authors:** Yongshun Wang, Jie Yuan, Huadong Liu, Jie Chen, Jieru Zou, Xiaoyi Zeng, Lei Du, Xin Sun, Zhengyuan Xia, Qingshan Geng, Yin Cai, Jingjin Liu

**Affiliations:** a Department of Cardiology, Shenzhen People's Hospital (The Second Clinical Medical College, Jinan University; The First Affiliated Hospital, Southern University of Science and Technology), Shenzhen, Guangdong 518020, China; b Shenzhen Clinical Research Center for Geriatrics, Shenzhen People’s Hospital, Shenzhen, Guangdong 518020, China; c Department of Anesthesiology, Affiliated Hospital of Guangdong Medical University, Zhanjiang, Guangdong 524001, China; d Faculty of Chinese Medicine, State Key Laboratory of Quality Research in Chinese Medicine, Macau University of Science and Technology, Taipa, Macao 999078, China; e Department of Health Technology and Informatics, The Hong Kong Polytechnic University, Kowloon, Hong Kong 999077, China

**Keywords:** AMPK, GLUT4, HDAC4, Heart failure, High intensity interval training, Meteorin-like protein

## Abstract

High-intensity interval training (HIIT) has been found to be more effective in relieving heart failure (HF) symptoms, than moderate-intensity continuous aerobic training (MICT). Additionally, higher meteorin-like protein (Metrnl) levels are seen after HIIT versus MICT. We investigated whether Metrnl contributed to post-HF cardiac functional improvements, and the signaling pathways involved. 50 HF patients underwent MICT, and another 50, HIIT, which was followed by cardiac function and serum Metrnl measurements. Metrnl was also measured in both blood and skeletal muscle samples of mice with transverse aortic constriction-induced HF after undergoing HIIT. Afterward, shRNA-containing adenovectors were injected into mice, yielding five groups: control, HF, HF + HIIT + scrambled shRNA, HF + HIIT + shMetrnl, and HF + Metrnl (HF + exogenous Metrnl). Mass spectrometry identified specific signaling pathways associated with increased Metrnl, which was confirmed with biochemical analyses. Glucose metabolism and mitochondrial functioning were evaluated in cardiomyocytes from the five groups. Both HF patients and mice had higher circulating Metrnl levels post-HIIT. Metrnl activated AMPK in cardiomyocytes, subsequently increasing histone deacetylase 4 (HDAC4) phosphorylation, leading to its cytosolic sequestration and inactivation via binding with chaperone protein 14-3-3. HDAC4 inactivation removed its repression on glucose transporter type 4, which, along with increased mitochondrial complex I-V expression, yielded improved aerobic glucose respiration and alleviation of mitochondrial dysfunction. All these changes ultimately result in improved post-HF cardiac functioning. HIIT increased skeletal muscle Metrnl production, which then operated on HF hearts to alleviate their functional defects, via increasing aerobic glucose metabolism through AMPK-HDAC4 signaling.

## Introduction

Heart failure (HF) comprises the end stage for various cardiac pathologies, such as ischemic and hypertensive heart diseases^1^; their risk factors (e.g., diabetes, coronary artery disease) are also shared with HF. Its five-year mortality rate is ∼40%,^2^^,^^3^ which, along with global population aging and adoption of sedentary lifestyles, has resulted in it being a significant health burden.^1^^,^^2^^,^^4^ Exercise has long been recommended for reducing HF likelihood and aiding in rehabilitation, and multiple studies have demonstrated higher exercise intensity and/or frequency being associated with lowered mortality and better quality of life.^4^, ^5^, ^6^ In particular, high-intensity interval training (HIIT), consisting of highly-intense exercises for <1–4 min at 85%–90% of peak oxygen uptake (VO_2peak_), interspersed with 5–10 min recovery periods at 50%–60% VO_2peak_,^7^^,^^8^ is considered a safe, effective post-HF rehabilitation regimen.^9^^,^^10^ Indeed, HIIT has been found to be more effective in alleviating HF symptoms and improving cardiac health, compared with the standard moderate-intensity continuous aerobic training (MICT), comprising consistent activity at 50%–60% VO_2peak_.^7^^,^^9^^,^^11^ However, the underlying bases for such improvements are still mainly unknown, though some studies found differences in protein expression levels, such as for meteorin-like protein (Metrnl), among MICT versus HIIT participants, as well as versus pre-exercise baseline levels.^12^, ^13^, ^14^, ^15^ However, its role in heart failure remains undefined.

Metrnl is a ∼28 kD protein, related to the neurotrophic growth factor meteorin.^12^^,^^13^ It is produced in both adipose tissue and skeletal muscle; in the latter, increased Metrnl mRNA has been observed post-exercise.^14^^,^^15^ Its precise functions have not been fully elucidated, though numerous studies have shown that it serves as an adipokine favoring fat metabolism, as well as an anti-inflammatory agent modulating immune responses.^12^^,^^16^ In the cardiovascular system, Metrnl alleviated HF symptoms in a mouse model, where its overexpression was associated with cardiac hypertrophy inhibition, while its knockdown increased the extent of cardiac tissue damage.^17^ The cardioprotective effect of Metrnl is facilitated via activating AMP-activated kinase (AMPK), which subsequently regulates histone-modifying protein activities, such as histone deacetylase 4 (HDAC4), to alter the expression of multiple genes.^18^^,^^19^ Normally, HDAC4 deacetylates histones to repress target gene expression^20^; however, AMPK activation results in HDAC4 phosphorylation (p-HDAC4) and sequestration within the cytoplasm, releasing this repression.^21^ One gene that HDAC4 represses is glucose transporter type 4 (GLUT4), which comprises ∼70% of all cardiomyocyte glucose transporters^22^^,^^23^ and facilitates glucose uptake. By contrast, insufficient energy supply is a major prerequisite of HF,^23^^,^^24^ as demonstrated in rat HF models which have lowered GLUT4 within left ventricular (LV) tissue, compared with wild type; this deficiency exacerbated maladaptive cardiac functional developments in hypertrophic hearts contributing to HF, particularly after exercise.^25^

Based on these findings, we postulated that HIIT may improve post-HF cardiac functioning by stimulating Metrnl expression, leading to AMPK activation and HDAC4 cytosolic sequestration, thereby increasing GLUT4 and glucose metabolism. In this study, we confirmed that HIIT is connected to Metrnl expression, where skeletal muscle produced higher Metrnl after HIIT, compared with MICT, in both human patients and mouse HF models. Higher Metrnl, in turn, increased AMPK activity, leading to HDAC4 phosphorylation and cytosolic sequestration, along with increased mitochondrial complex I–V expression. P-HDAC4 sequestration released GLUT4 repression, leading to improved aerobic glucose respiration and mitochondrial function among HF hearts. Therefore, Metrnl could serve as a possible therapeutic approach for alleviating post-HF functional defects.

## Methods and materials

### Study population

During March–July 2021, 100 HF patients with mid-range EF, defined as having 40%–49% LV ejection fraction (LVEF) and meeting New York Heart Association Classes II-III HF criteria, despite receiving treatment for ≥12 weeks in accordance with American Heart Association guidelines, were randomly recruited at the Department of Cardiology, Shenzhen People's Hospital. Exclusion criteria were second/third-degree atrioventricular block, ventricular arrhythmia history, recent (<4 weeks prior to study start) unstable angina, myocardial infarction or coronary revascularization, uncontrolled HF, significant renal/hepatic disease, severe chronic pulmonary obstructive disease (COPD) or aortic stenosis, acute pulmonary embolism/myocarditis, symptomatic cerebrovascular disease within 12 months, or expected mortality in ≤12 months. All patients provided written informed consent. The study was approved by the Committee for Medical and Health Ethics of Shenzhen People's Hospital, Jinan University (REB #: LL-KY-2021154).

### HIIT and MICT exercise protocols

Patients were randomly divided into two groups; one did MICT as control, and the other HIIT, on a stationary bicycle (using an ergometer SCHILLER CS-200) over 12 weeks, for three sessions per week. MICT consisted of continuous cycling at 45%–60% of heart rate reserve (HRR), for 30–45 min, while HIIT comprised four 4-min intervals at 75%–80% of HRR, with active pauses of 3 min of cycling at 45%–50% of HRR between each interval. 90/100 were able to complete all 36 HIIT/MICT assigned stationary bicycle exercises (Fig. S1); their characteristics were described in Table S1. Echocardiography was performed for both groups, at baseline and immediately after 12 weeks (Table 1), while cardiopulmonary parameters, expressed as VO_2peak_, were measured at 2 days before (baseline), and 2 days after the 12 weeks by a single rehabilitation physician blinded to patient exercise conditions. All patients performed the graded exercise test, and VO_2peak_ results were shown in Table S2, in which VO_2peak_ was significantly higher in HIIT, indicating it was more intense than in MICT (Table S3). Safety for both MICT and HIIT was defined in terms of significant adverse events, as provided in Table S2.

**Table 1:** Echocardiography measurements of cardiac functional parameters from patients at baseline and after a 12-week exercise period.

Cardiac functional parameter measurements						
Parameter	MICT (*n* = 48)			HIIT (*n* = 42)		
	Baseline	12 weeks	*P*	Baseline	12 weeks	*P*
LVEDD (mm)	53.65 (52.45–54.84)	52.10 (51.23–52.98)	0.0385∗	54.79 (53.59–55.98)	52.98 (52.12–53.83)	0.0149∗
LVEF (%)	44.8 (44.0–45.6)	46.31 (45.4–47.3)	0.0156∗	44.7 (43.8–45.6)	47.2 (46.1–48.3)	0.0004∗
NT-proBNP (ng/L)	1171 (1033–1308)	757.0 (671.6–842.3)	<0.0001∗	1082 (916.5–1248)	544.1 (463.8–624.5)	<0.0001∗
Serum Metrnl	207.3 (178.5–236.1)	246.1 (214.1–278.1)	0.0726	191.3 (157.7–224.9)	286.6 (240.8–332.5)	0.0011∗

Changes in cardiac functional parameters from baseline to after the 12-week exercise period			
Parameter	MICT (*n* = 48)	HIIT (*n* = 42)	*P* (MICT *vs*. HIIT)
LVEDD (mm)	−1.542 (−2.125 to −0.958)	−1.810 (−2.418 to −1.201)	0.5245
LVEF (%)	1.521 (1.013–2.029)	2.548 (1.756–3.339)	0.0266∗
NT-proBNP (ng/L)	−413.8 (−500.9 to −326.7)	−538.2 (−679.1 to −397.3)	0.1236
Serum Metrnl	38.81 (20.46–57.17)	95.33 (63.95–126.7)	0.0017∗

Data are presented as medians (95% confidence interval). ∗*P* < 0.05 is considered statistically significant.

### Mouse transverse aortic constriction (TAC)-induced HF model

Animal studies were approved by the Animal Care and Ethics Committee of Jinan University, following the Guide for the Care and Use of Laboratory Animals from the National Institutes of Health. Male C57BL/6 mice (8–10 weeks) were anesthetized with ketamine/xylazine (80 mg/kg + 10 mg/kg), followed by TAC, via tying a suture around the transverse aorta, over a 27-gauge blunted needle. Sham-operated mice served as controls. All mice were closely monitored for 4 weeks.

### HIIT exercise training for mice

Four weeks after TAC, mice were initially acclimated to the treadmill for HIIT, 2 weeks prior to the 12-week assessment period. Mice's endurance capabilities were measured by their receiving a 60 s warm-up at 5 m/min, followed by treadmill running in 20 s intervals, beginning at 7 m/min, then 10 m/min, and subsequently increasing by 1 m/min. Each 20 s interval had a 20 s relative rest period (treadmill belt at 5 m/min) in between. Exercises ended upon mouse exhaustion, signified by their either touching the shock grid 3 times or suffering from 7 shocks in total. Mice were then subjected to an uphill performance test, which served as the basis for HIIT exercises, based on a similar protocol as the endurance capability measurement test, but on a 20° inclined treadmill. Uphill performance tests were carried out both at the beginning, as well as after 5 weeks into the 12-week HIIT period, to re-adjust training intensity.

Mouse HIIT consists of high-intensity interval uphill training, comprising 13 alternations during 4 min of running on a 20° inclined treadmill, at an intensity corresponding to 85%–90% of the maximum speed obtained during the uphill performance test, followed by 2 min rest. To acclimatize the mice, exercise intensity was gradually increased over the first 2 weeks, from a 0°–20° treadmill incline. HIIT was administered 5 days per week, for a duration of 12 weeks.

### Pressure-volume (P-V) loop assessment

Mice were anesthetized, and a P-V loop catheter was inserted into the right common carotid artery. LV function, including end-systolic (ESV) and diastolic volumes (EDV), as well as maximum and minimum pressure change rate (±dP/dt) and LVEF, was determined when the catheter was advanced into the LV chamber. Data analysis was carried out by LABSCRIBE2 (iWorx System).

### *In vivo* micro-dialysis of electrically-stimulated mouse gastrocnemius muscle contractions

To obtain dialysate from electrically-stimulated mouse gastrocnemius muscle, the skin was opened down the posterior aspect of 1 hindlimb, and an LM10 micro-dialysis probe was inserted longitudinally into the gastrocnemius muscle. The proximal part of the hindlimb was immobilized using 2 pins, and the foot was fixed securely to an upright post. The distal tendon of the gastrocnemius muscle was tied, cut, and attached to a force transducer (model 50-7915, Harvard Apparatus) to record the isometric contractile force. The resulting signals were amplified using a Harvard transducer amplifier and recorded by a Lectromed MX216 recorder. To provide electrical stimulation, an electrode was placed onto the sciatic nerve and connected to the stimulator (model S48, Grass Instruments), and the skin was closed after completing the surgical procedures. The micro-dialysis probe was perfused at 4 μL/min, for 90 min, using a fluid with a similar composition to the interstitial fluid; afterward, the dialysate was collected every 10 min in an ice-cooled vial.

### Serum Metrnl measurements using ELISA

Blood samples were collected from both patients and mice immediately after exercise and centrifuged at 3000 rpm for 10 min at 4 °C. Serum Metrnl concentration was measured using human or mouse Metrnl ELISA kits (R&D systems), according to the manufacturer's instructions.

### Isolation of primary cardiomyocytes and treatment

To isolate cardiomyocytes, mice were anesthetized and administered heparin (0.5 mL heparin/mouse, 100 IU/mL, LEO Pharma) to prevent coagulation. Hearts were collected and immediately perfused with perfusion buffer and then digested by infusing digestion buffer containing collagenase II (2.4 mg/mL, Thermo Fisher Scientific). After digestion, heart tissue was minced, and myocytes mechanically dispersed and filtered. The rod-shape primary cardiomyocytes were maintained on Matrigel-coated culture plates in Eagle's Minimum Essential Medium (Thermo Fisher Scientific), supplemented with 0.1% bovine serum albumin (Sigma–Aldrich), 1% penicillin/streptomycin, and 2 mM glutamine (Sigma–Aldrich), and cultured for 1 h at 37 °C within a humidified atmosphere containing 5% CO_2_. Some subgroups of those primary cardiomyocytes were treated with or without 10 μmol/L Compound C (Sigma Aldrich).

### Western blotting

Total protein samples (80 μg) were subjected to electrophoresis in 4%–15% SDS-PAGE gel, followed by transfer into polyvinylidene difluoride membranes, which were then blocked with 5% casein at room temperature for 1 h. Primary antibody incubation for Metrnl (Cat # ab235775, Abcam), GLUT4 (Cat # 2213, Cell Signaling Technology), phosphorylated AMPK (p-AMPK; Cat # 2537, Cell Signaling Technology), AMPK (Cat # 2532, Cell Signaling Technology), p-HDAC4 (Cat # 3424, Cell Signaling Technology), HDAC4 (Cat # 15164, Cell Signaling Technology), Histone H3 (Cat # 4499, Cell Signaling Technology), glyceraldehyde-3-phosphate dehydrogenase (GAPDH; Cat # 2118, Cell Signaling Technology), β-Tubulin (Cat # 2146, Cell Signaling Technology), α-porin (Cat # ab14734, Abcam), and mitochondrial complex antibody cocktail (Cat # ab110413, Abcam) was carried out (all 1:1000) at 4 °C overnight. Afterward, the membranes were incubated with horseradish peroxidase-conjugated secondary antibody (1:2000) at room temperature for 90 min. After being washed 3 times, membranes were then subjected to enhanced chemiluminescence detection (WesternBright). Images were captured using the ChemiDoc XRS system (Biorad) and analyzed by ImageJ.

### Histological and immunofluorescent staining of cardiac and skeletal muscle tissue sections

LV tissue was fixed in 4% paraformaldehyde, embedded in paraffin, and sectioned into 5 μm sections, which were stained with Sirius red staining reagents (Cat # ab150681, Abcam). Collagen content was measured by ImageJ. To examine mitochondrial morphology, 100 nm sections were stained with uranyl acetate and Reynold's lead citrate, followed by transmission electron microscope photography (CM100; Philips Electron Optics). Numbers of mitochondria per field were presented, based on the average of at least 5 fields.

For immunofluorescence staining, cardiomyocytes or skeletal muscle tissue were fixed in 4% paraformaldehyde, blocked with 10% goat serum, and then incubated with anti-mouse Metrnl (1:100; Cat # bs-18810R, Bioss) or p-HDAC4 (1:100; Cat # bs-10328R, Bioss). Cells/tissues were then incubated with their respective secondary antibodies, imaged with a Carl Zeiss LSM 780 confocal microscope, and analyzed using ImageJ.

### HDAC4 immunoprecipitation and chromatin immunoprecipitation (ChIP)

Total protein was extracted from mouse cardiomyocytes, using lysis buffer and a freshly-added protease inhibitor tablet (Roche). Immunoprecipitation was performed by first incubating with anti-14-3-3 antibody (Abcam, ab97273) at 4 °C overnight, followed by incubation with Protein A/G agarose beads at 4 °C for 4 h. Afterwards, protein A/G beads were washed 6 times with phosphate buffer saline, and proteins of interest were eluted with SDS loading buffer. The interaction of 14-3-3 with p-HDAC4 was confirmed via immunoblotting with anti-p-HDAC4 antibody (Cat # 3424, Cell Signaling Technology), anti-HDAC4 (Cat # 15164, Cell Signaling Technology), and anti-GAPDH (Cat # 2118, Cell Signaling Technology) antibodies at 4 °C overnight.

A commercially-available kit was used for ChIP, following the manufacturer's instructions (Cell Signaling Technology). Briefly, mouse heart tissue was minced, and proteins cross-linked to DNA by 1% formaldehyde at room temperature for 20 min, followed by adding glycine (125 mM) to stop the cross-linking reaction. The tissue was then lysed in a lysis buffer supplemented with a protease inhibitor cocktail from the kit, followed by sonication to generate DNA fragments. Afterward, the total tissue lysates were subjected to immunoprecipitation with protein A/G magnetic beads, in the presence of primary HDAC4 antibody (1:200) or mouse IgG control, at 4 °C overnight, to obtain the DNA/protein complexes. These complexes, after washing by lysis buffer, were then eluted with elution buffer, and subsequently incubated at 62 °C for 2 h to free the DNA. DNA purification using commercially-available spin columns (Merck), following the manufacturer's instructions, and the resulting DNA fragments were analyzed by real-time quantitative PCR using specific GLUT4 primers: forward 5′-CTTCGACCTTTCAGGGGAC-3′ and reverse 5′-GAACAAAAGGCTCTTCCCGC-3′, as HDAC4 has been found in the literature to interact with GLUT4^44^.

### Measuring mitochondrial reactive oxygen species (ROS) levels, membrane potential, and complex I–V enzyme activity

ROS levels in mitochondria were measured by staining cells with MitoSox Red (0.5 μM, excitation/emission 510/580 nm, Thermo Fisher, M36008). Superoxide levels were determined based on changes in MitoSOX Red fluorescence under a Carl Zeiss LSM 780 confocal microscope. Image analysis was performed with ImageJ.

The level of ΔΨm, representing mitochondrial membrane potential, was determined by the JC-1 mitochondrial membrane potential assay kit (Thermo Fisher), following the manufacturer's instructions.

Activity levels for mitochondrial complexes I–V were measured using commercially available enzyme assays, in accordance with the manufacturer's instructions (Cat # ab109721, ab109905, ab109911, ab109714; Abcam). Cardiac tissues were manually homogenized in mitochondrial isolation buffer (MIB; 250 mmol/L sucrose, 0.5 mmol/L Na_2_EDTA, 10 mmol/L Tris, and 0.1% BSA at pH 7.4), using a medium-fitting glass Teflon Potter-Elvehjem homogenizer. The resulting homogenate was centrifuged twice at 1000 g for 5 min each to obtain the supernatant, which, in turn, was centrifuged twice at 11,000 g for 10 min to yield a mitochondrial pellet that was collected and re-suspended in 3 times the pellet volume of MIB. Mitochondrial pellets from MIB were then re-suspended in phosphate buffer saline, and supplemented with 10% detergent from the kits to obtain lysates, and the protein concentrations in the lysates were measured thereafter. Twenty-five (for complex I, IV, and V) or 100 μg (for complex II + III) of mitochondrial protein was used for each reaction in their appropriate assays. Enzyme activities were measured with a spectrophotometer in triplicate and expressed as changes in absorbance per minute per mg protein.

### Glucose uptake measurements

Glucose uptake was measured using the 2-deoxy-d-glucose uptake measurement kit (Cat # ab136955, Abcam). Cardiomyocytes were starved for 2–4 h, followed by the addition of 2-deoxy-d-glucose and incubation at 37 °C for 20 min. Afterward, cells were washed with phosphate buffer saline, lysed with extraction buffer, and repeatedly pipetted. Cell lysates were then frozen in liquid nitrogen, followed by being heated at 85 °C for 40 min. The resulting supernatant was collected, and glucose uptake was measured, following the manufacturer's instructions.

### Analysis of oxidative and glycolytic metabolic rates

Oxygen consumption (OCR) and extracellular acidification (ECAR) rates for cardiomyocytes were measured using, respectively, the seahorse XF Mito and glycolysis stress test kits on an XF24 extracellular flux analyzer (Agilent Technologies). Cells were plated 1 day prior to metabolic analysis, and metabolic rates were measured in the absence (basal conditions) or presence of inhibitors. For OCR, the following compounds were sequentially added into the cells through different ports in the flux analyzer: glucose (5 mM), oligomycin (ATP synthase inhibitor; 1 μM), carbonyl cyanide 4-(trifluoromethoxy) phenylhydrazone (mitochondrial uncoupling agent FCCP; 1 μM), and rotenone/antimycin A (mitochondrial complex inhibitor; 1 μM), while for ECAR, instead of FCCP and rotenone/antimycin A, 2-deoxy-glucose (2-DG, glucose analog serving as a hexokinase inhibitor; 5 mM) was added after glucose and oligomycin. The resulting OCR and ECAR were normalized to basal respiration rates.

### Adenovirus-mediated overexpression and knockdown of Metrnl

To knock down endogenous Metrnl, mice were intravenously injected with adenoviral vectors containing short hairpin RNA (shRNA) against Metrnl (shMetrnl; targeting sequences 5′-CACGCTTTAGTGACTTTCAAA-3′), while vectors containing scrambled shRNA (Src-shRNA) served as the control (Hanbio Biotechnology, Shanghai, China). Both adenovirus vector types were prepared and titrated to 10^11^ transfection units/mL.

Metrnl overexpression was obtained *in vitro* by treating cardiomyocytes with 200 ng/mL Metrnl, while *in vivo*, mice were intraperitoneally injected with recombinant mouse Metrnl protein, at 30 μg/day per mouse, once every 2 days for 12 weeks (R&D systems).

### Nanoscale liquid chromatography with tandem mass spectrometry (NANO-LC-MS/MS) analysis

To prepare proteins for nano-LC-MS/MS, 200 μg of proteins for each sample were incorporated into 30 μL SDT buffer (4% SDS, 100 mM DTT, 150 mM Tris-HCl at pH 8.0). Repeated ultrafiltration with UA buffer (8 M urea, 150 mM Tris-HCl at pH 8.5) was then used to remove SDS, DTT, and other low-molecular-weight (≤30 kD) components (Sartorius). Reduced cysteine residues were blocked by adding 100 μL iodoacetamide (IAA; 100 mM in UA buffer) to prevent disulfide bond formation, and the samples were incubated in the dark for 30 min, followed by filtration. Filters were washed 3 times with 100 μL UA buffer and then 2 times with 100 μL 0.1 M triethylammonium bicarbonate (TEAB) buffer. Afterward, protein suspensions were digested at 37 °C overnight with 4 μg trypsin (Promega), in 40 μL 0.1M TEAB, and the resulting peptides were collected as a filtrate. Peptide content was determined by measuring UV absorbance at 280 nm, using an extinction coefficient of 1.1 of 0.1% (g/L) solution, which was based on tryptophan and tyrosine frequencies in vertebrate proteins. After measuring the peptide content in the filtrates, 100 μg of each sample was labeled using tandem mass tag reagent, according to the manufacturer's instructions (Thermo Fisher Scientific).

Nano-LC-MS/MS was carried out by injecting each fraction for analysis. Specifically, peptide mixtures were loaded onto the C18-reversed phase analytical column (Thermo Fisher) in buffer A (0.1% formic acid) and then separated with a linear gradient of buffer B (80% acetonitrile and 0.1% formic acid) at a flow rate of 300 nL/min. MS was then performed on a Q Exactive Plus mass spectrometer (Thermo Fisher), in positive ion mode, which was coupled to Easy nLC for nano-LC (Thermo Fisher Scientific). MS data was acquired using a data-dependent top10 method, involving dynamic selection of the most abundant precursor ions from the survey scan (350–1800 *m*/*z*) for higher-energy C-trap dissociation fragmentation. The automatic gain control target was set to 3e6, and the maximum injection time to 45 ms. Survey scans were acquired at a resolution of R = 70,000 at m/z 200, resolution for HCD spectra was R = 17,500 at m/z 200, and isolation width was 2 *m*/*z*. The normalized collision energy was 30 eV. Proteins found to have significant differences between control and model groups were considered differentially expressed proteins (DEPs), which were identified using the Database for Annotation Visualization and Integrated Discovery. Biological process, cellular component, and molecular function were annotated by the Gene Ontology (GO) database, while the signaling pathways for identified proteins were elucidated by searching against the Kyoto Encyclopedia of Genes and Genomes (KEGG) database.

### Statistical analysis

Statistical analyses were performed by SPSS ver. 20.0. The data were expressed as mean ± standard deviation (SD), except for baseline demographic information between MICT and HIIT patients, which were presented as mean values with 95% confidence intervals (CI), or as percentages. These groups were compared by either Student's *t*-test or Mann–Whitney *U* test for continuous variables, and chi-squared test for nominal variables. Pearson correlation determined associations between Metrnl with either EF or blood N-terminal pro-brain natriuretic peptide (NT-proBNP). Student's *t*-test was used for comparisons between 2 groups, while one-way analysis of variance (ANOVA), followed by Bonferroni *post hoc* tests, was used for 3 or more groups for all other analyses. *P* < 0.05 was considered statistically significant.

For Nano-LC-MS/MS, proteins with *P* < 0.05 and fold change ≥ ±1.8 were considered DEPs. False discovery rates were controlled using the Benjamini-Hochberg procedure. GO and KEGG analyses were carried out using Fisher's exact test, with the entire database of quantified protein annotations serving as the background dataset. Only categories and pathways with *P* < 0.05 were considered statistically significant.

## Results

### HIIT is associated with increased serum Metrnl and improved cardiac functioning

One hundred HF patients, defined as having 40%–49% LVEF, were recruited for this study (Fig. S1). The 48 patients in the MICT group and 42 in the HIIT group who completed the exercises were examined after the 12-week exercise period, and no significant differences in clinical characteristics or significant adverse events were found, except for ischemic cardiomyopathy, previous myocardial infarction history, COPD, or the use of diuretics (Table S1, 2).

**Figure 1:**
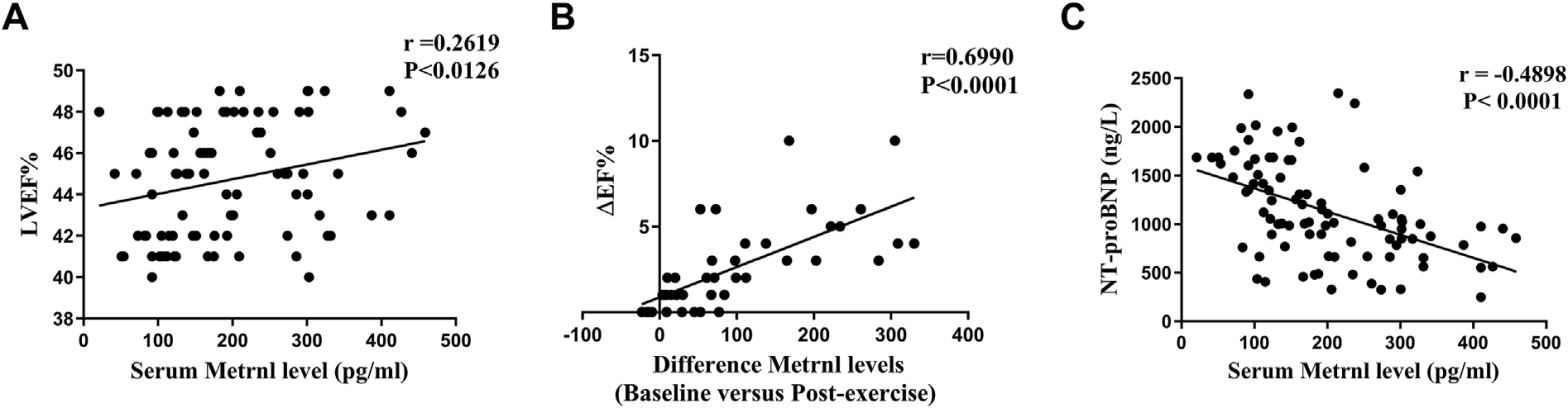
Increased serum meteorin-like protein (Metrnl) levels are associated with improved cardiac functional parameters among heart failure (HF) patients after the 12-week exercise period. **(A)** The scatter plots from HF patients demonstrating the presence of a positive correlation between increased serum Metrnl levels and left ventricular ejection fraction (LVEF), a measure of cardiac function, as well as **(B)** between changes in serum Metrnl and changes of LVEF. **(C)** A negative correlation is present between serum Metrnl and NT-proBNP, a marker of HF.

With respect to cardiac function, the HF marker NT-proBNP, and Metrnl, patients in both MICT and HIIT groups had significantly decreased LV end-diastolic diameter (LVEDD) and NT-proBNP, as well as increased LVEF, after the 12-week exercise period, compared with baseline. However, the levels of LVEDD and NT-proBNP decreases, as well as the extent of LVEF increase, were significantly greater in the HIIT group than in the MICT group, likely owing to the significant increase of blood Metrnl seen in the former compared with the latter (Table 1). Indeed, increased Metrnl was positively associated with LVEF and negatively associated with NT-proBNP under Pearson correlation (Fig. 1A–C). All these findings collectively indicate that although both MICT and HIIT could improve cardiac functioning, HIIT yielded significant increases in serum Metrnl and thus was more able to augment these exercise-associated cardiac functional improvements post-HF.

### Metrnl increased during skeletal muscle contraction and alleviated post-HF cardiac functioning in a mouse model

**Figure 2:**
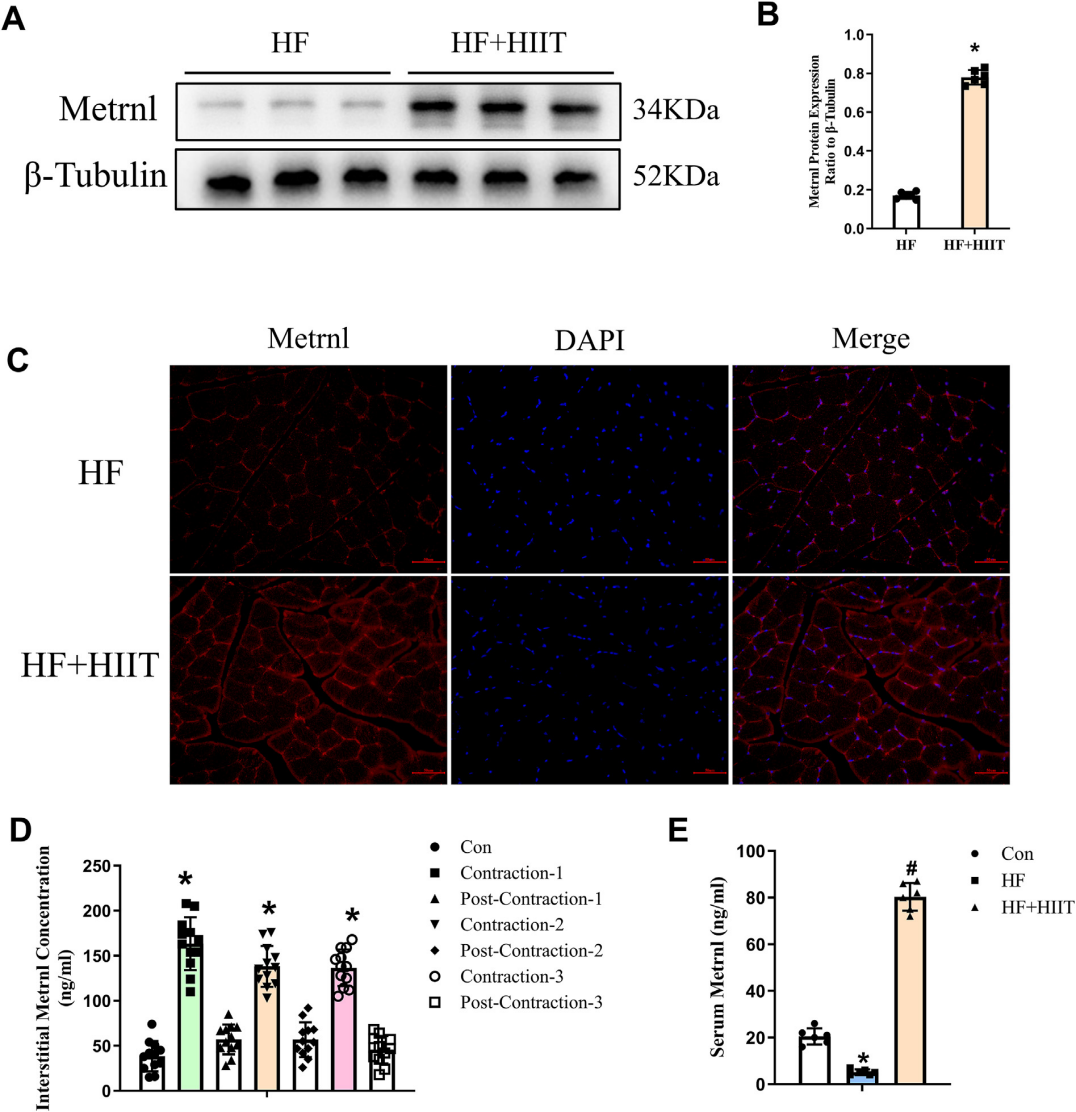
Metrnl expression and secretion from skeletal muscle increased under high-intensity interval training (HIIT) in an HF mouse model. **(A, B)** Western blot analysis showing increased Metrnl expression, normalized to tubulin, in hindlimb muscles among HF (transverse aortic constriction-induced) mice who underwent HIIT exercises (HF + HIIT), compared with HF alone. **(C)** Representative immunofluorescence images demonstrating increased gastrocnemius muscle Metrnl expression among HF + HIIT versus HF groups. Scale bar, 50 μm. **(D)***In vivo* interstitial micro-dialysis results from electrically-stimulated mouse gastrocnemius muscle demonstrating that muscle contraction (Contraction-1, -2, -3) increases Metrnl secretion. Post-contraction (Post-Con 1, -2, and -3) periods were 20 min each, to allow for muscle recovery between contraction periods. **(E)** Serum Metrnl levels between sham-treated control (Con), HF, and HF + HIIT groups. The data were shown as mean ± SD. *n* = 6 mice/group for all experiments, ^∗^*P* < 0.05 *vs*. Con or Post-Con, ^*#*^*P* < 0.05 *vs*. HF.

To further elucidate the link between HIIT and increased Metrnl, we examined whether skeletal muscle contraction was involved in Metrnl expression in a TAC-induced HF mouse model. Western blot demonstrated that HF mice subjected to HIIT had higher hind-limb muscle Metrnl, compared with non-HIIT (Fig. 2A, B), which was further supported by immunofluorescent staining, where Metrnl was localized within skeletal muscle fibers, and had higher expression levels among HF + HIIT versus HF alone (Fig. 2C). Micro-dialysis from electrically-stimulated gastrocnemius muscle also showed that increased Metrnl expression, manifesting as increased interstitial Metrnl, directly corresponded to muscle contraction; contraction-1, -2, and -3 phases had the highest levels, compared with non-contracting control, as well as post-contraction phases (Post-Con 1, 2, and 3; Fig. 2D). We next examined whether Metrnl was secreted from skeletal muscle into the circulation, and found that serum Metrnl decreased in HF, compared with sham control (Con); however, HF + HIIT reversed this decrease (Fig. 2E). These results thus indicate that gastrocnemius and hindlimb muscle contractions during HIIT stimulated skeletal muscular Metrnl synthesis and secretion into the bloodstream.

**Figure 3:**
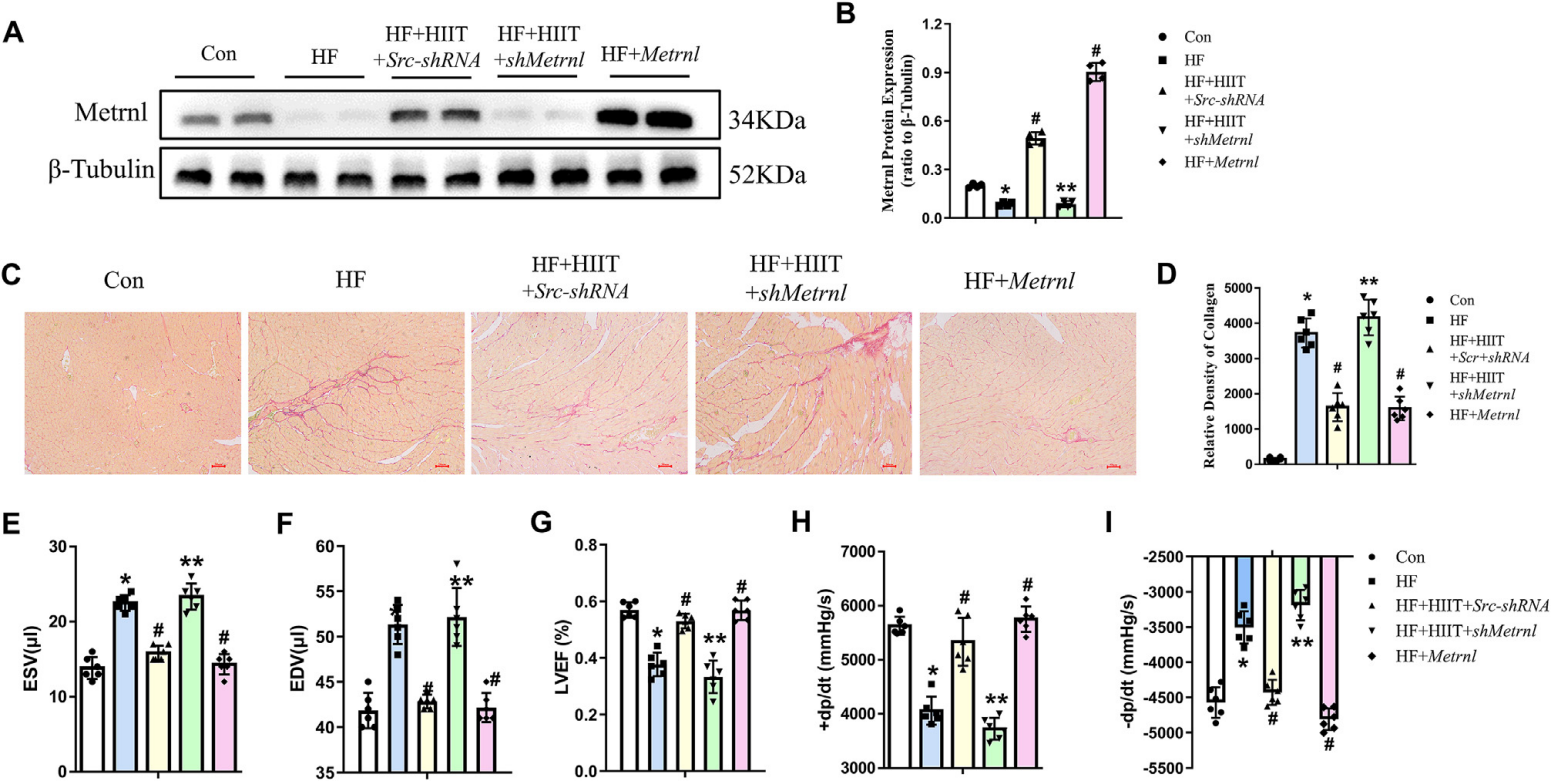
Increased Metrnl levels alleviated cardiac functional parameters in HF mice, which were all abrogated upon Metrnl knockdown. **(A, B)** Western blot analysis of cardiac Metrnl levels, normalized to tubulin, in Con, HF, HF + HIIT + Src-shRNA (HF with HIIT and scrambled short hairpin RNA), HF + HIIT + shMetrnl (HF with HIIT and Metrnl knockdown by shRNA), and HF + Metrnl (HF with exogenous Metrnl) groups. **(C)** Representative Sirius Red collagen staining and **(D)** quantification of collagen content for histological cardiac tissue sections among the 5 groups. Scale bar: 20 μm. Pressure-volume loop assessments among the 5 groups for **(E)** end-systolic (ESV) and **(F)** diastolic volumes (EDV), as well as **(G)** left ventricular ejection fraction (LVEF, %), **(H)** + dP/dt, and **(I)** −dP/dt (changes in blood pressure during contraction). The data were shown as mean ± SD. *n* = 4 mice/group for (A, B), *n* = 6 mice/group for (C–I). ∗*P* < 0.05 *vs*. Con, ^*#*^*P* < 0.05 *vs*. HF, ∗∗*P* < 0.05 *vs*. HF + HIIT + Src-shRNA.

We then examined whether the association of increased Metrnl with improved post-HF cardiac function among HIIT patients was also present in the mouse model. Five mice groups were examined: Con, HF, HF + HIIT + Src-shRNA, HF + HIIT + shMetrnl, and HF + Metrnl. Adenovirus vectors containing either Src-shRNA or shMetrnl were intravenously injected into mice pre-HIIT respectively, in HF + HIIT + Src-shRNA and HF + HIIT + shMetrnl groups, while in HF + Metrnl group, intraperitoneal administration of exogenous Metrnl achieved Metrnl overexpression. Subsequent Western blot results showed that compared with Con, HF had significantly lower Metrnl, while the level of Metrnl was significantly increased in HF + HIIT + Src-shRNA compared with HF. However, the HIIT-associated increase in Metrnl expression seen in the HF + HIIT + Src-shRNA group was abolished by shMetrnl, yielding Metrnl at similar levels to that of HF. This reversal further supports the association between HIIT and increased Metrnl. Additionally, HF + Metrnl had the highest Metrnl (Fig. 3A, B). Metrnl expression patterns among the 5 groups also corresponded to differences in cardiac tissue collagen extent under Sirius staining, a myocardial fibrosis indicator, in which HF mice had significantly more collagen than Con. By contrast, this post-HF collagen increase was partially reversed towards Con levels in HF + HIIT + Src-shRNA group, as well as in HF + Metrnl group; the highest amount of collagen, though, was present in the HF + HIIT + shMetrnl group. Therefore, increased Metrnl was able to reduce post-HF myocardial fibrosis (Fig. 3C, D).

P-V loop assessment was conducted to evaluate cardiac function, where HF mice, compared with Con, had significantly increased ESV (Fig. 3E) and EDV (Fig. 3F), as well as lower LVEF (Fig. 3G). With respect to ± dP/dt, HF had lower + dP/dt (Fig. 3H) and higher −dP/dt (Fig. 3I). These changes, however, were reversed back towards Con levels for all parameters among HF + HIIT + Src-shRNA and HF + Metrnl. On the other hand, for all parameters, HF + HIIT + shMetrnl had similar levels to HF, bolstering the association between increased Metrnl and post-HF cardiac functional improvements. All evidence thus supports that HIIT stimulated Metrnl synthesis and secretion from skeletal muscle into the circulation. Metrnl, in turn, operates upon the heart to counteract HF pathological developments, such as fibrosis, ultimately leading to improved cardiac functionality.

### Cardiomyocytes from HF mice subjected to HIIT have different protein expression profiles from those without HIIT

**Figure 4:**
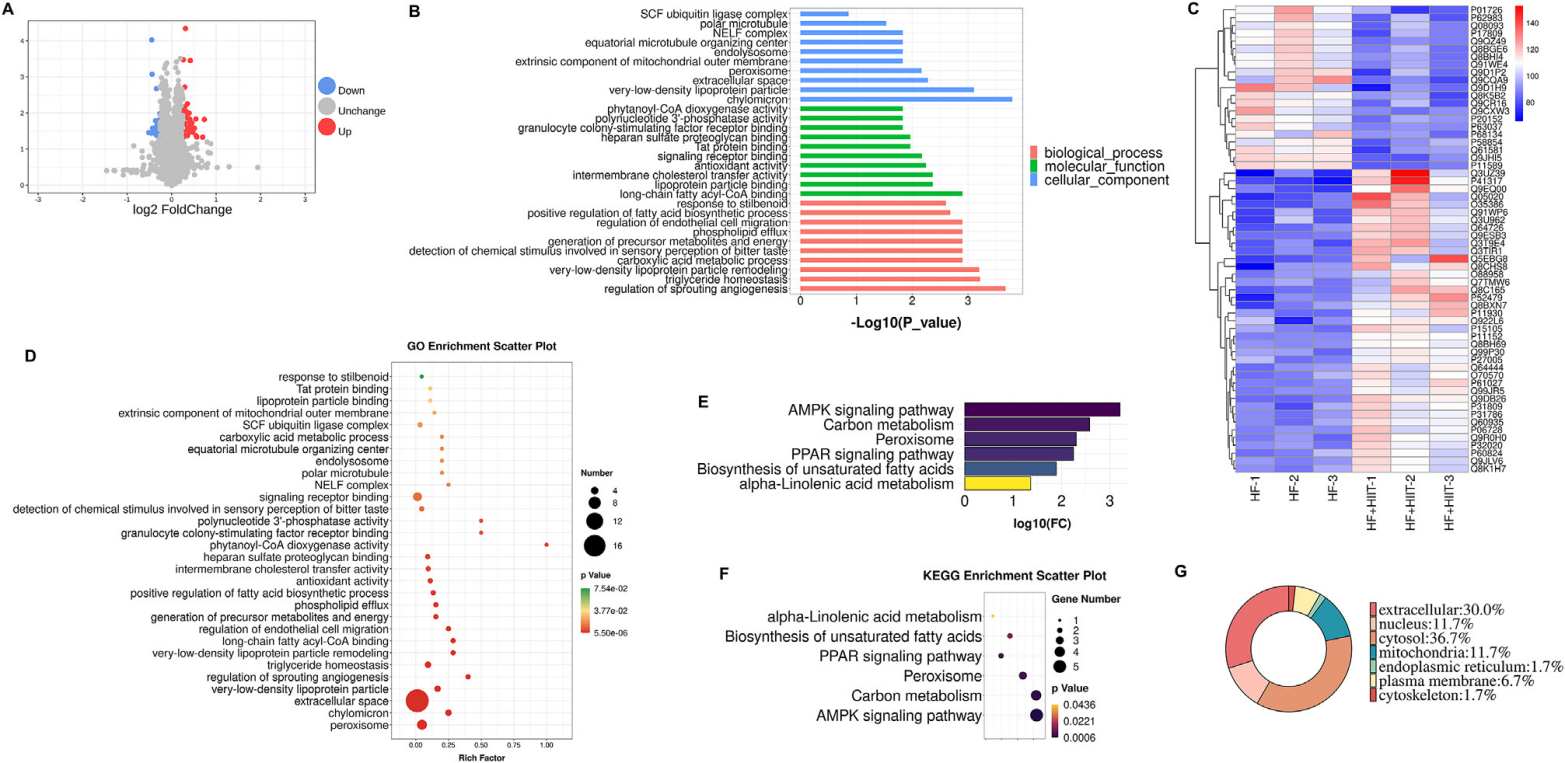
Gene ontology (GO) and Kyoto Encyclopedia of Genes and Genomes (KEGG) analyses of differentially-expressed proteins (DEPs) between HF- versus HF + HIIT-derived cardiomyocytes. **(A)** Volcano plot showing proteins that are up- (red) and down-regulated (blue) in HF + HIIT mice, compared with HF alone. **(B)** The 60 DEPs fall under 3 broad categories: cellular component, molecular function, and biological process, with 10 subcategories within each category. **(C)** The heat map showing the 60 differentially-expressed proteins (DEPs) between the 2 groups, with up to a 2-fold change in expression, of which 39 were up- (red) and 21 down-regulated (blue). **(D)** GO enrichment analyses of the 60 DEPs, verifying the categorization of these DEPs into those 3 categories and 30 subcategories. KEGG analyses, in the form of **(E)** histogram and **(F)** scatter plots, showing the top 6 pathway associations for the 60 DEPs. **(G)** Distribution of the 60 DEPs in terms of subcellular localization. *n* = 3 mice/group, *P* < 0.05 for HF + HIIT *vs*. HF.

To shed light on the underlying mechanisms responsible for HIIT-stimulated Metrnl to alleviate HF, we performed nano-LC-MS/MS on cardiomyocytes isolated from HF mouse hearts, with or without HIIT. As shown in the volcano plot, a number of proteins either had increased or decreased expression levels among HF + HIIT mice, compared with HF alone (Fig. 4A); more specifically, 39 proteins were up-regulated, and 21 down-regulated in HF + HIIT versus HF, with at least 2-fold change in expression levels (Fig. 4C). These 60 DEPs fell within 3 broad categories: cellular component, molecular function, and biological process, each of which contained 10 subcategories (Fig. 4B). Among the 30 subcategories in the 3 groups, 10 were associated with lipid metabolism, and 2 with glucose metabolism, indicating that a significant portion of DEPs is associated with the regulation of metabolism (Fig. 4B). DEP correspondence with these 30 subcategories was also supported by GO analysis (Fig. 4D). To further elucidate the pathways these DEPs are involved in, KEGG analysis was used, in which out of the ∼126 analyzed pathways, the top 6 were AMPK signaling (5 proteins), carbon metabolism (4), peroxisome (3), PPAR signaling (2), biosynthesis of unsaturated fatty acids (2), and alpha-linolenic acid metabolism (1) (Fig. 4E, F), further supporting the association of a significant portion of the proteins with energy-related metabolism under GO. Additionally, subcellular location analysis showed that DEPs were predominantly expressed in the cytoplasm (36.7%) and extracellularly (30%) (Fig. 4G). Overall, HIIT exercises in HF mice were associated with cardiomyocyte protein expression profiles favoring increased energy-based metabolism, particularly with respect to glucose and lipids, compared with non-HIIT.

### Increased Metrnl under HIIT favors aerobic glucose metabolism, along with increased p-AMPK and GLUT4

**Figure 5:**
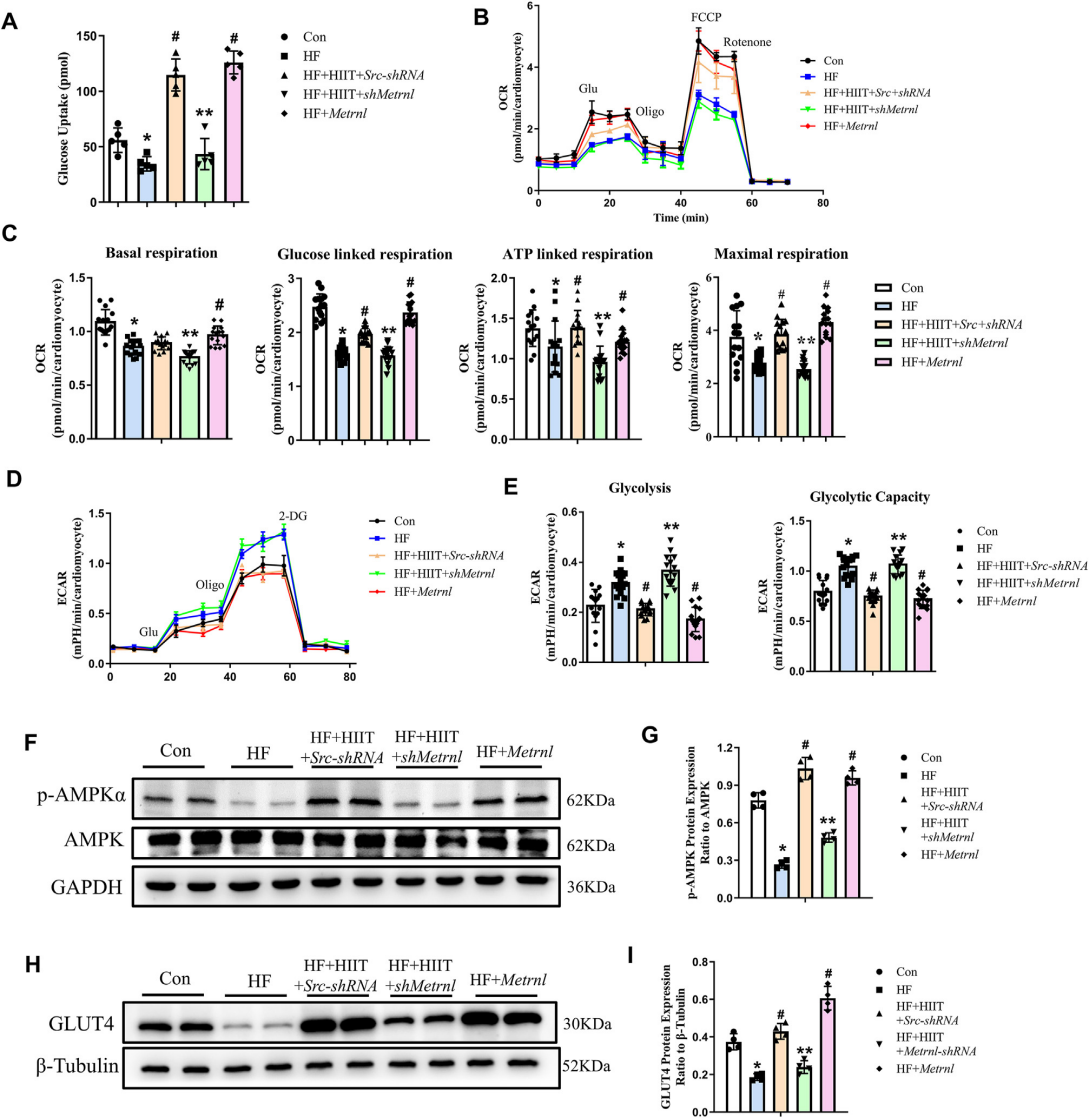
Increased Metrnl levels promoted aerobic glucose metabolism among cardiomyocytes by increasing p-AMPK and GLUT4 expression. **(A)** Measurements of glucose uptake among Con, HF, HF + HIIT + Src-shRNA, HF + HIIT + shMetrnl, and HF + Metrnl groups. **(B)** Representative traces of oxygen consumption rate (OCR) measurements for all 5 groups. **(C)** Quantification of basal, glucose-linked, ATP-linked, and maximal respiration rates, derived from OCR measurements, for all 5 groups. **(D)** Representative traces of extracellular acidification rate (ECAR) measurements. **(E)** Quantification analysis of glycolysis and glycolytic capacity, derived from ECAR measurements, for all 5 groups. **(F, G)** Western blot analysis of AMP-activated protein kinase (AMPK) and phosphorylated AMPK (p-AMPK) expression levels, normalized to GAPDH, among the 5 groups. **(H, I)** Western blot analysis of glucose transporter type 4 (GLUT4), normalized to tubulin, among the 5 groups. The data were presented as mean ± SD. *n* = 5 mice/group for (A, D, E), *n* = 15 mice/group for (B, C), and *n* = 4 mice/group for (F–I). ^∗^*P* < 0.05 *vs*. Con, ^#^*P* < 0.05 *vs*. HF, ^∗∗^*P* < 0.05 *vs*. HF + HIIT + Src-shRNA.

To determine whether Metrnl was associated with increased metabolism-associated gene expression in HF mice subjected to HIIT, as predicted by GO and KEGG, we measured glucose uptake in cardiomyocytes isolated from the 5 treatment groups. We found that compared with Con, HF had lower glucose uptake, while HF + HIIT + Src-shRNA had significantly higher levels than the former two groups. However, the rate of glucose uptake in the HF + HIIT + shMetrnl group was similar to that of HF. This observation, along with the fact that HF + Metrnl has uptake levels similar to that of HF + HIIT + Src-shRNA, indicates that increased glucose uptake among HF mice after HIIT is attributable to HIIT-stimulated increase of Metrnl (Fig. 5A). The impact of HIIT and Metrnl expression on subsequent glucose metabolism was then examined by measuring OCR, an aerobic glucose metabolism indicator, and ECAR, a glycolytic flux parameter. All 5 groups demonstrated similar OCR patterns upon glucose, oligomycin, FCCP, and rotenone/antimycin A administration, representing, respectively, glucose-linked, ATP-linked, and maximal respiration measurements (Fig. 5B). However, within those 5 groups, for glucose-linked, ATP-linked, and maximal respiration, HF and HF + HIIT + shMetrnl consistently had significantly lower OCRs, compared with Con, while HF + HIIT + Src-shRNA and HF + Metrnl had levels comparable to Con. Therefore, increased Metrnl is able to alleviate the negative impact of HF on glucose metabolic processes by bolstering aerobic respiration (Fig. 5C). This also corresponds with lowered anaerobic respiration, or glycolysis, represented by ECAR, in which all 5 groups shared similar patterns upon glucose administration, representing glycolysis, as well as oligomycin and 2-deoxyglucose, representing glycolytic capacity (Fig. 5D). However, unlike OCR, ECAR was significantly higher for both glycolysis and glycolytic capacity in HF and HF + HIIT + shMetrnl, compared with Con (Fig. 5E), indicating that HF favors anaerobic respiration, in light of HF-associated cell damage negatively affecting aerobic metabolism. This increase, however, is reversed back towards Con levels among HF + HIIT + Src-shRNA and HF + Metrnl, demonstrating that HIIT could reduce the need for glycolysis by supporting more efficient pro-aerobic respiration among cardiomyocytes (Fig. 5E). This is achieved through increasing Metrnl, demonstrating its involvement in glucose metabolic processes.

To confirm Metrnl's involvement with increasing aerobic versus anaerobic glucose metabolism, particularly with respect to the AMPK pathway indicated by KEGG, Western blot was used to further investigate the interactions between Metrnl and AMPK. We found that while AMPK levels remained the same among all 5 groups, activated p-AMPK was significantly lower among HF and HF + HIIT + shMetrnl, compared with Con. This decrease, however, was reversed among HF + HIIT + Src-shRNA and HF + Metrnl, indicating that increased Metrnl resulted in AMPK pathway activation (Fig. 5F, G). Next, we examined GLUT4 expression, a glucose transporter reported to be the downstream p-AMPK target. Indeed, GLUT4 was significantly lower in HF and HF + HIIT + shMetrnl than Con, while it was comparable or higher in HF + HIIT + Src-shRNA and HF + Metrnl (Fig. 5H, I). All these results indicated that Metrnl operates through the AMPK pathway to alleviate the detrimental effects of HF on aerobic cardiac metabolism, possibly through GLUT4, in turn lessening the need for anaerobic processes.

### Metrnl fosters increased glucose metabolism by increasing cytosolic p-HDAC4

**Figure 6:**
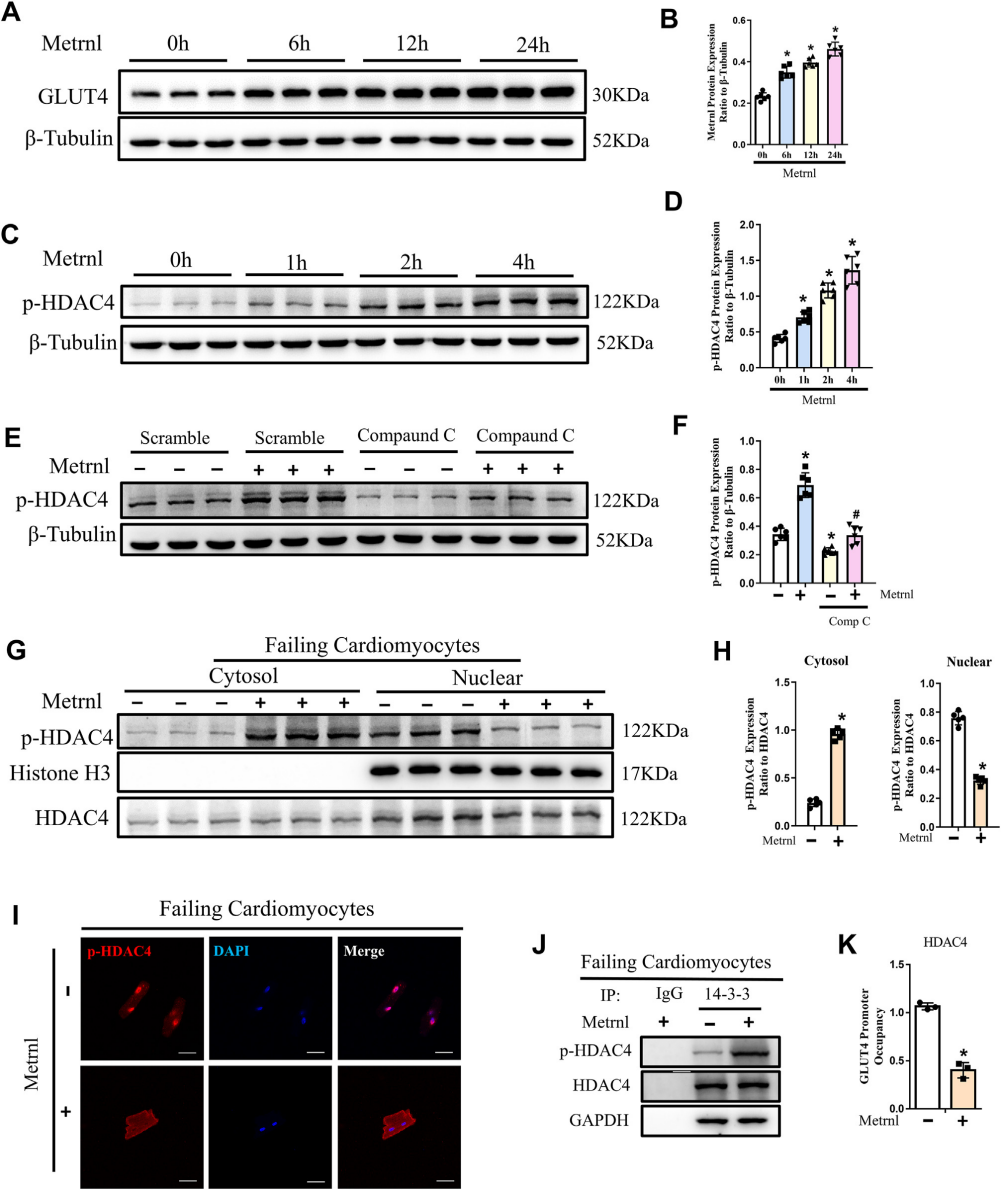
Increased Metrnl resulted in increased p-HDAC4 and cytoplasmic sequestration. **(A**, **B)** Western blot analysis of GLUT4 levels, normalized to tubulin, over 24 h of Metrnl treatment in HF-derived cardiomyocytes. **(C**, **D)** Western blot analysis of p-HDAC4 levels, normalized to tubulin, over 4 h of Metrnl treatment. **(E**, **F)** Western blot analysis of p-HDAC4 levels, normalized to tubulin, from HF cardiomyocytes treated with/without Metrnl and/or compound C (AMPK inhibitor). **(G**, **H)** Western blot analysis of p-HDAC4 and HDAC4 levels, as well as sequestration within either the nucleus or cytosol, with/without Metrnl. **(I)** Representative immunofluorescence images of p-HDAC4 localization within HF cardiomyocytes. Scale bar, 50 μm. **(J)** Western blot of co-immunoprecipitation results for p-HDAC4 and 14-3-3 chaperone protein, with/without Metrnl. **(K)** Chromatin immunoprecipitation results regarding HDAC4 binding to the GLUT4 promoter, with/without Metrnl. The data were presented as mean ± SD. *n* = 5 mice/group for (A, B, G, H), *n* = 6 mice/group for (C–F), and *n* = 3 mice/group for (J, K). ^∗^*P* < 0.05 *vs*. 0 h for (B, D) or *vs*. without Metrnl in (F, H, K).

HDAC4 has been documented to act on the GLUT4 promoter to repress its expression.^26^ However, it is deactivated via phosphorylation and subsequent interaction with chaperone protein 14-3-3 for cytoplasmic retention, thereby releasing its repression of GLUT4.^27^ To determine whether Metrnl had any impact on p-HDAC4 expression, and in turn on GLUT4, we treated HF mouse cardiomyocytes with Metrn1 over a period of 24 h for investigating GLUT4 expression, and 4 h for p-HDAC4. Western blot showed that Metrnl increased both GLUT4 (Fig. 6A, B) and p-HDAC4 (Fig. 6C, D) in a time-dependent manner. To further elucidate Metrnl and p-HDAC4 interactions, particularly AMPK pathway involvement, we administered the selective AMPK inhibitor, compound C, prior to Metrnl treatment, and found that compared with untreated control, compound C significantly decreased p-HDAC4. However, even with compound C, Metrnl increased p-HDAC4 towards those found in the control, suggesting that Metrnl may also regulate p-HDAC4 via alternative mechanisms (Fig. 6E, F). This p-HDAC4 increase also involved alterations in its intracellular distribution, where Metrnl administration in HF cardiomyocytes favored p-HDAC4 localization within the cytosol, rather than the nucleus, while the opposite was the case for cells without Metrnl (Fig. 6G, H). This change was supported by p-HDAC4 immunofluorescence staining (Fig. 6I). p-HDAC4 cytosolic sequestration was also supported by 14-3-3 and p-HDAC4 co-immunoprecipitation; there, Metrnl was associated with increased co-precipitation of 14-3-3 with p-HDAC4, and thus greater cytosolic retention (Fig. 6J). Lastly, ChIP found that Metrnl treatment led to decreased HDAC4 binding to GLUT4 promoters within HF cardiomyocytes, thereby increasing GLUT4 levels (Fig. 6K). All these data suggest that increased Metrnl, likely via p-AMPK and/or another putative pathway, up-regulated GLUT4 by sequestrating p-HDAC4 within the cytoplasm.

### Metrnl improved mitochondrial function via increasing complex I–V expression and aerobic respiration

**Figure 7:**
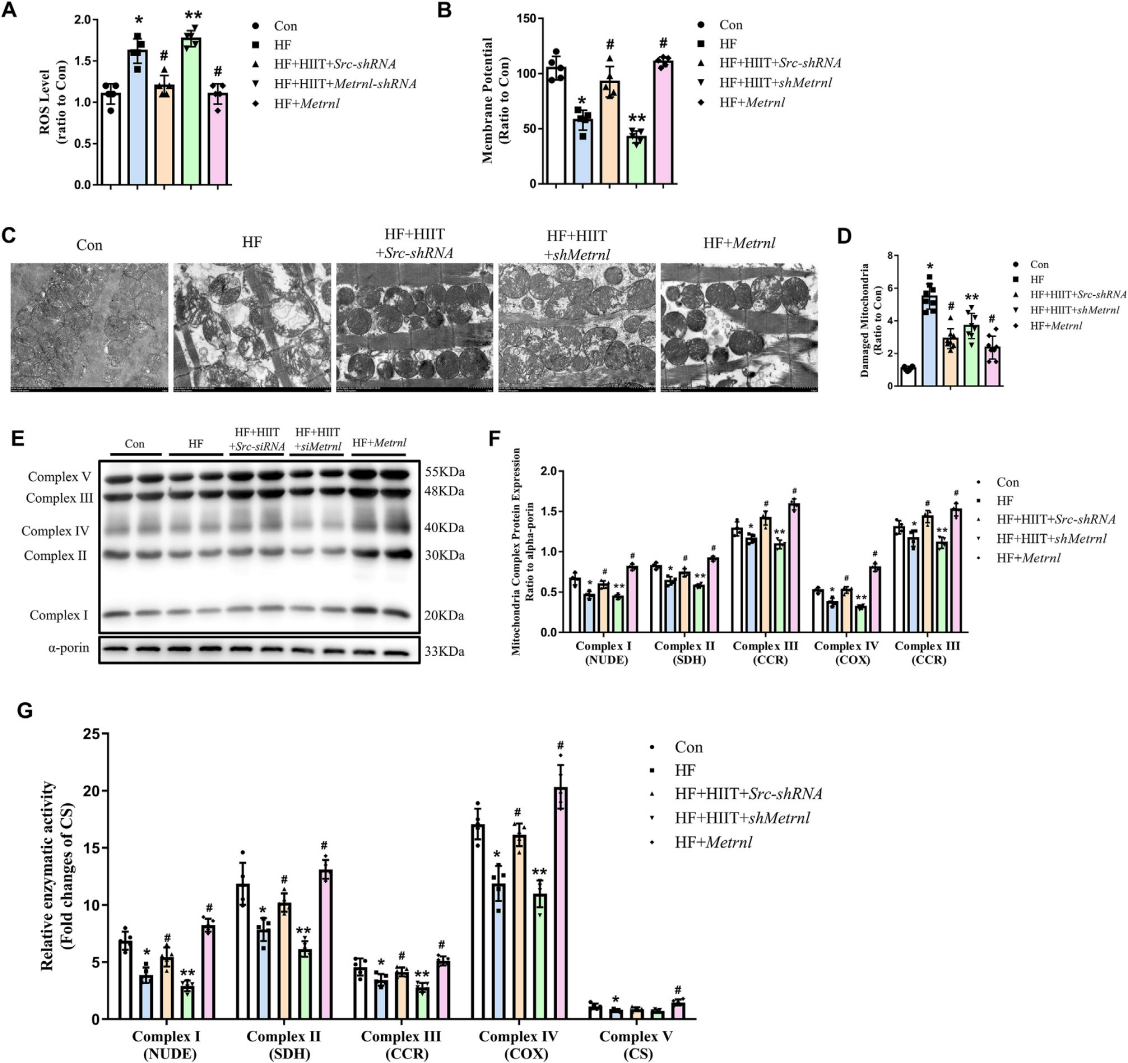
Increased Metrnl reversed post-HF mitochondrial structural damage, functioning, as well as complex I–V protein expression and activity. **(A)** Reactive oxygen species (ROS) levels among Con, HF, HF + HIIT + Src-shRNA, HF + HIIT + shMetrnl, and HF + Metrnl cardiomyocyte groups. **(B)** Mitochondrial membrane potential measurements among the 5 groups. **(C)** Representative transmission electron microscope micrographs depicting cardiomyocyte mitochondrial morphological changes between the five groups (magnification 8000 × ). Scale bar, 2 μm. **(D)** Counts of damaged mitochondria among the five groups. **(E**, **F)** Western blot analysis of mitochondrial complex I–V protein expression levels among the five groups. **(G)** Measurements of complex I–V enzymatic activity among the five groups. The data were presented as mean ± SD. *n* = 5 mice/group for (A, B, G), *n* = 8 mice/group for (D), and *n* = 4 mice/group for (F). ^∗^*P* < 0.05 *vs*. Con, ^#^*P* < 0.05 *vs*. HF, ^∗∗^*P* < 0.05 *vs*. HF + HIIT + Src-shRNA.

Lowered glucose aerobic metabolism has often been associated with decreased mitochondrial functioning. To determine if that was the case in HF mouse cardiomyocytes, as well as to examine the impact of Metrnl on mitochondrial function, we measured ROS levels, which have long been associated with mitochondrial dysfunction.^28^ As expected, HF was associated with significantly higher ROS, compared with Con. However, HF + HIIT + Src-shRNA reduced ROS back towards those seen in Con, while HF + HIIT + shMetrn1 increased ROS towards levels comparable to HF (Fig. 7A). All these results indicated that HIIT reduced ROS via increasing Metrnl expression, suggesting that Metrnl may alleviate mitochondrial dysfunction. These findings were also supported by similar results found with mitochondrial membrane potential, where compared with Con, HF and HF + HIIT + shMetrnl had lowered levels, while HF + HIIT + Src-shRNA and HF + Metrnl were able to restore membrane potential to similar levels as Con (Fig. 7B). Furthermore, transmission electron microscope images showed that HF cardiomyocytes had significantly more abnormally-shaped mitochondria with poorly-defined cristae, characteristic of mitochondrial damage, compared with Con (Fig. 7C, D), though damaged mitochondria counts were reduced towards Con among HF + HIIT + Src-shRNA and HF + Metrnl (Fig. 7D). To identify the underlying molecular basis, we investigated mitochondrial complex I–V expression levels, involved in oxidative phosphorylation, among the five groups. Western blot results showed that for all five complexes, protein levels decreased in HF and HF + HIIT + shMetrnl, compared with Con, which was reversed back towards Con levels or even higher in HF + HIIT + Src-shRNA and HF + Metrnl, indicating that increased Metrnl is associated with increased complexes I–V (Fig. 7E, F). Complex I–IV enzymatic activity followed the same pattern (Fig. 7G). All these findings indicate that Metrnl is able to alleviate mitochondrial damage and dysfunction in HF cardiomyocytes, by increasing complex I–V protein expression and enzymatic activity, and subsequently bolstering aerobic glucose metabolism.

## Discussion

Exercise has been observed to improve post-HF cardiac functioning, though its specific underlying basis has not been fully defined. However, one possible mechanism may be Metrnl, whose skeletal muscular expression has been observed to increase upon exercise. Here, we confirmed that exercise stimulated increased Metrnl, and in turn improved cardiac functional and tissue structural parameters, among both HF patients and a mouse model of HF. Furthermore, HIIT triggered greater Metrnl production, compared with standard MICT, among those patients. The connection between HIIT, Metrnl, and improved post-HF cardiac functioning was also demonstrated by exogenous Metrnl administration yielding comparable functional and myocardial tissue improvements to that of HIIT; by contrast, Metrnl knockdown reversed these improvements. Increased Metrnl, in turn, was associated with increased AMPK, mitochondrial complex I–V, p-HDAC4, and GLUT4, as well as restoring proper mitochondrial morphologies. All these changes thus substantiate the association of Metrnl with glucose metabolism, found under GO and KEGG, which is also demonstrated by the presence of improved mitochondrial function and aerobic metabolic activity upon increased Metrnl, in the form of being able to reverse the post-HF decrease in OCR, as well as the increase in ECAR and ROS production. This reversal serves as the basis for improved cardiac function.

Numerous studies have shown a connection between exercise and improved cardiac function, in terms of lowering HF risk and alleviating post-HF functional defects.^29^ For instance, Hambrecht et al demonstrated that endurance training reversed HF-associated LV remodeling and modestly improved LVEF, which were substantiated by subsequent long-term studies.^30^^,^^31^ Based on these findings, MICT has been recommended as part of HF rehabilitation strategies.^7^^,^^29^ However, HIIT has become increasingly popular recently, as some studies have shown that its greater intensity, compared with MICT, yields greater improvements in cardiac functioning and survival.^7^, ^8^, ^9^^,^^11^^,^^32^^,^^33^ These studies, however, have been contradicted by others indicating that little difference is present between HIIT and MICT, particularly among HF patients with preserved cardiac fraction.^34^ Our study, though, is in line with the aforementioned literature demonstrating greater cardiac functional improvements among HF patients with HIIT, compared with MICT. We also found that this difference is likely owed to the greater stimulation of Metrnl production under HIIT, which is in line with studies demonstrating exercise-induced Metrnl up-regulation in both mice and human skeletal muscles.^15^^,^^35^

Metrnl is a protein secreted from white adipose tissue, skeletal muscle, and immune cells, which has been found to play various immune-related and metabolic roles.^12^^,^^13^^,^^15^^,^^16^^,^^36^ In particular, it plays a role in skeletal muscle regeneration, via Stat3 activation, to recruit anti-inflammatory macrophages, which then secrete IGF-1 to activate satellite cells.^37^ Furthermore, Metrnl also increases skeletal muscle glucose metabolism by increasing intracellular Ca^2+^, leading to greater AMPKα2 activity.^19^ Increased AMPKα2, in turn, led to greater HDAC5 phosphorylation, increasing its sequestration within the cytosol via increasing p-HDAC5-14-3-3 interactions, and thereby increasing GLUT4 expression by reducing p-HDAC5 repression.^19^ This result from Lee et al, intriguingly, is similar to what we observed in our study with respect to cardiac tissue, where increased Metrnl led to more AMPK activity and increased p-HDAC4, yielding increased GLUT4 expression and subsequent glucose metabolism. These mechanistic parallels between skeletal and cardiac tissue, with respect to Metrnl impact on glucose metabolism, were further supported by Jiang et al, where in an HF mouse model, exercise activated AMPK, resulting in increased p-HDAC4 and GLUT1, along with improved cardiac functioning and lowered mitochondrial structural damage.^38^ Indeed, HDAC4 inhibition was associated with improved post-HF cardiac functioning, while GLUT1 knockdown impaired this, further validating the pathway found in our studies between AMPK, HDAC4, and GLUT4.^38^

HDAC4 has been found to play a role in both skeletal and cardiac muscles, in which its inhibition favors improved muscle functioning.^39^^,^^40^ More specifically, HDAC4 overexpression exacerbated post-myocardial infarction cardiac dysfunction, remodeling, and interstitial fibrosis, owing to lowered heart cardiokine levels.^40^^,^^41^ Metrnl is likely one of those cardiokines, as knocking out Metrnl yielded similar outcomes to that of HDAC4 overexpression, as shown by Ruperez et al, entailing increased cardiac remodeling post-cardiac hypertrophy induction.^17^ By contrast, Metrnl overexpression prevented cardiac remodeling,^17^ owing to AMPK activation, as demonstrated in another study where Metrnl overexpression alleviated cardiomyocyte apoptosis post-ischemia/reperfusion.^18^ In agreement with those findings, our study also demonstrated a connection between increased Metrnl, HDAC4 inhibition, and improved cardiac functioning.

There are a number of limitations within this study, though, one of which was that a small sample size of 100 HF patients, only comprising those with mid-range EF, was investigated. Furthermore, whether HIIT could improve myocardial microcirculation was not examined. Additionally, the extent of heart injury, as represented by troponin I, or high-sensitivity troponin I levels, was not monitored after exercises, thus, whether MICT or HIIT could cause minor myocardial damage is still an open question. Patients were also only examined during 12 weeks, so any deleterious impacts from long-term HIIT, such as possible hypertrophy and development of arrhythmia, have still not been fully elucidated. Therefore, future studies should examine the long-term safety and efficacy of HIIT, with respect to possible improvements in microcirculation, or conversely, in causing minor myocardial damage.

## Conflict of interests

The authors declare that there is no conflict of interests.

## Funding

This study was supported by the 10.13039/501100001809National Natural Science Foundation of China (No. 82200315), 10.13039/501100021171Guangdong Basic and Applied Basic Research Foundation of China (No. 2021A1515111145), Sanming Project of Medicine in Shenzhen, Guangdong, China (No. SZSM201412012), Science and Technology Planning Project of Shenzhen Municipality, Guangdong, China (No. JCYJ20210324113807021, JCYJ20210324113614038), Major scientific research project of Shenzhen People's Hospital (China) (No. SYWGSJCYJ202301), and Life Science Research Start-up Fund of PolyU SZRI (China) (No. I2021A008). All authors have reported that they have no relationships relevant to the contents of this paper to disclose.
