# Supplementary material for: Elevated meteorin-like protein from high-intensity interval training improves heart function via AMPK/HDAC4 pathway
Source: Genes Dis. 2023 Sep 14;11(6):101100. doi: 10.1016/j.gendis.2023.101100 (PMC11400619; doi:10.1016/j.gendis.2023.101100)
Supplement: Multimedia component 1 [file mmc1.docx]

**Supplemental Material**

for

**Elevated meteorin-like protein from high-intensity interval training improves heart function via AMPK/HDAC4 pathway**

Yongshun Wang^a,b#^, Jie Yuan^a,b#^, Huadong Liu^a,b^, Jie Chen^a,b^, Jieru Zou^a,b^, Xiaoyi Zeng^a,b^, Lei Du^a,b^, Xin Sun^a,b^, Zhengyuan Xia ^c,d^, Qingshan Geng^a,b*^, Yin Cai^e*^, Jingjin Liu^a,b*^

^a^Department of Cardiology, Shenzhen People's Hospital (The Second Clinical Medical College, Jinan University;The First Affiliated Hospital, Southern University of Science and Technology), Shenzhen 518020, Guangdong,China;

^b^Shenzhen Key Laboratory of Stem Cell Research and Clinical Transformation，Shenzhen People’s Hospital (The Second Clinical Medical College,Jinan University;The First Affiliated Hospital, Southern University of Science and Technology), Shenzhen 518020, Guangdong, China;

^c^Department of Anesthesiology, Affiliated Hospital of Guangdong Medical University, Zhanjiang 524001, Guangdong, China;

^d^Faculty of Chinese Medicine, State Key Laboratory of Quality Research in Chinese Medicine, Macau University of Science and Technology, Taipa, Macao, China;

^e^Department of Health Technology and Informatics, The Hong Kong Polytechnic University, Hong Kong SAR, China.

^#^These authors contributed equally to this work.

*ADDRESS FOR CORRESPONDENCE:

Jingjin Liu and Qingshan Geng, Department of Cardiology, Shenzhen People's Hospital, Shenzhen, China, or Yin Cai, Department of Health Technology and Informatics, The Hong Kong Polytechnic University, Hong Kong SAR, China. Email: liu.jingjin@szhospital.com, gengqsh@163.net OR david-yin.cai@polyu.edu.hk.

**Supplemental Figure 1**


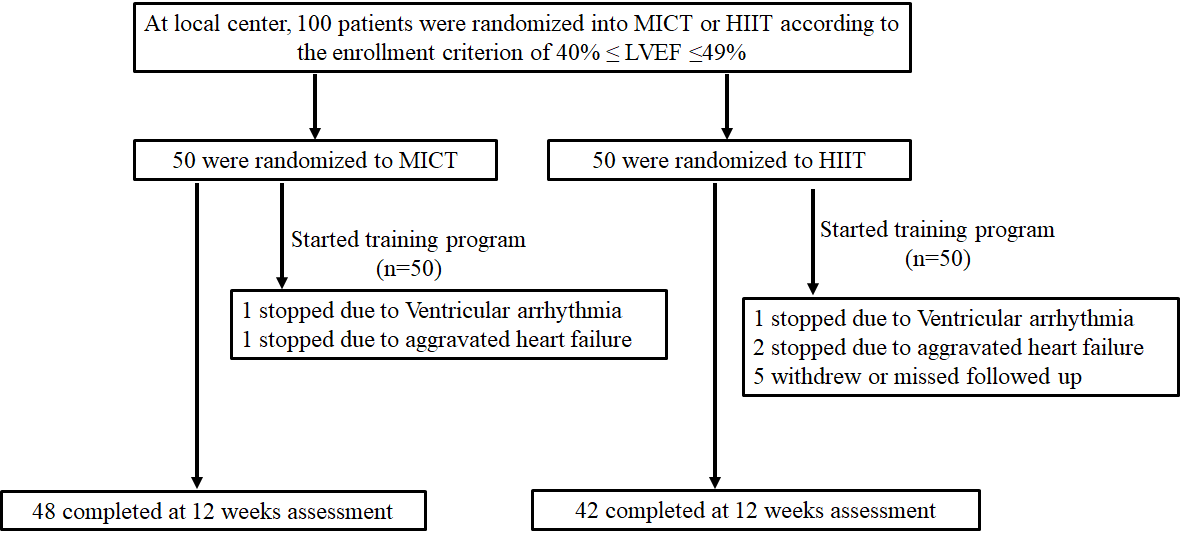


**SUPPLEMENTAL FIGURE 1** Flowchart of moderate intensity continuous aerobic (MICT) and high intensity interval training (HIIT) exercise groups of heart failure (HF) patients. Out of the 100 patients initially enrolled into the study, 90 were able to complete all 36 assigned stationary bicycle exercises at the end of the 12-week assessment period, which were used as the basis for all subsequent analyses.

**Supplemental Tables**

**SUPPLEMENTAL TABLE 1** Patient characteristics at baseline before exercise

| Patient characteristic | MICT (n=48) | HIIT (n=42) | P |
| --- | --- | --- | --- |
| Age | 63.1 (60.6-65.6) | 63.8 (61.3-66.4) | 0.6859 |
| Female (%) | 18 (37.5%) | 15 (35.71%) | 0.8627 |
| Current smoker ( n [%]) | 31 (64.58%) | 25 (59.52%) | 0.6260 |
| Body mass index (kg/m^2^) | 23.75 (23.06-24.43) | 23.66 (22.78-24.54) | 0.8728 |
| Systolic blood pressure (mm Hg) | 145.4 (136.7-154.0) | 143.2 (133.1-153.3) | 0.7377 |
| Diastolic blood pressure (mm Hg) | 86.31 (82.59-90.04) | 84.64 (81.64-87.65) | 0.4922 |
| Heart rate | 78.60 (75.36-81.85) | 82.05 (78.58-85.51) | 0.1472 |
| NT-proBNP (ng/L) | 1171 (1033-1308) | 1082 (916.5-1248) | 0.4062 |
| NYHA III (%) | 22 (45.8%) | 17 (40.4%) | 0.6136 |
| LVEF (%) | 44.8 (44.0-45.6) | 44.7 (43.8-45.6) | 0.8340 |
| Ischemic cardiomyopathy | 30 (62.5%) | 39 (92.85%) | *0.0005 |
| Previous myocardial infarction | 25 (52.08%) | 32 (76.19%) | *0.0177 |
| Previous PCI | 28 (58.33%) | 32 (76.19%) | 0.0744 |
| Previous CABG | 2 (4.17%) | 0 (0%) | 0.1562 |
| Atrial fibrillation | 11 (22.91%) | 7 (16.67%) | 0.4652 |
| Pacemaker | 1 (2.08%) | 0 (0%) | 0.3525 |
| Hypertension | 27 (56.25%) | 29 (69.05%) | 0.2160 |
| Diabetes Mellitus | 21 (43.75%) | 19 (45.23%) | 0.8888 |
| COPD | 14 (29.17%) | 21 (50%) | *0.0436 |
| β-Blocker | 48 (100%) | 42 (100%) | 1 |
| ACEI or ARB | 48 (100%) | 42 (100%) | 1 |
| Aldosterone receptor antagonist | 45 (93.75%) | 39 (92.85%) | 0.8673 |
| Diuretic | 41 (85.42%) | 28 (66.67%) | *0.0362 |
| Statin | 42 (87.5%) | 40 (95.23%) | 0.2024 |
| Serious Adverse Events | 4 (8.33%) | 6 (14.28%) | 0.3757 |
| Plasma Metrnl (pg/ml) | 207.3 (78.5-236.1) | 191.3 (157.7-224.9) | 0.4660 |
| VO_2peak_ (mL/kg/min) | 16.33 (15.48-17.17) | 16.31 (15.41-17.22) | 0.9947 |

All values represent medians (95% CI), except otherwise noted. **P*<0.05 was statistically significant.

ACEI, angiotensin-converting enzyme inhibitor; ARB, angiotensin receptor blocker; CABG, coronary artery bypass graft; CI, confidence interval; COPD, chronic obstructive pulmonary disease; HIIT, high intensity interval training; LVEF, left ventricular ejection fraction; MICT, moderate intensity continuous aerobic interval training; NT-proBNP, blood N-terminal pro-brain natriuretic peptide; NYHA III, New York Heart Association class III; PCI, percutaneous coronary intervention; VO_2peak_, peak oxygen uptake.

**SUPPLEMENTAL TABLE 2** Occurrence of serious adverse events (SAEs) during the 12-week exercise period

| SAEs | MICT (n=48) | HIIT (n=42) |
| --- | --- | --- |
| Fatality | 0 | 0 |
| Life-threatening ventricular arrhythmia | 0 | 0 |
| Non-fatal ventricular arrhythmia | 2 (4.17%) | 3 (7.1%) |
| Acute heart failure | 0 | 0 |
| Worsening heart failure | 1 (2.08%) | 2 (4.76%) |
| Other non-fatal cardiac event | 1 (2.08%) | 1 (2.38%) |
| Total | 4 (8.33%) | 6 (14.28%) |

**SUPPLEMENTAL TABLE 3** Peak oxygen uptake (VO_2peak_) at Baseline and 12 weeks after the exercise period

| VO_2peak_ measurements | | | | | | | | |
| --- | --- | --- | --- | --- | --- | --- | --- | --- |
|  | | MICT (n=48) | | | HIIT (n=42) | | | |
|  | | Baseline | 12 weeks | P | Baseline | | 12 weeks | P |
| VO_2peak_ (mL/kg/min) | | 16.33 (15.48-17.17) | 17.42 (16.47-18.37) | 0.0854 | 16.31 (15.41-17.22) | | 18.54 (17.53-19.54) | *0.0013 |
| Changes in VO_2peak_ from baseline to after the 12-week exercise period | | | | | | | | |
|  | MICT (N=48) | | HIIT (N=42) | | | P value (MICT vs. HIIT) | | |
| VO_2peak_ (mL/kg/min) | 1.096 (0.9133-1.278) | | 4.246 (2.733-5.759) | | | *<0.0001 | | |

All values represent medians (95% CI). **P* <0.05 was statistically significant.
